# Factors Associated with Increased Burden of Caregivers of People with Dementia with Lewy Bodies

**DOI:** 10.3390/geriatrics9050115

**Published:** 2024-09-09

**Authors:** Shunji Toya, Mamoru Hashimoto, Yuta Manabe, Hajime Yamakage, Manabu Ikeda

**Affiliations:** 1 Medical Science, Sumitomo Pharma Co., Ltd., Tokyo 103-6012, Japan; 2 Department of Psychiatry, Osaka University Graduate School of Medicine, Osaka 565-0871, Japan; 3 Department of Neuropsychiatry, Kindai University Faculty of Medicine, Osakasayama 577-8502, Japan; 4 Department of Advanced Clinical Medicine, Division of Dementia and Geriatric Medicine, Kanagawa Dental University School of Dentistry, Yokosuka 238-0003, Japan; 5 Insight Clinical Development Group, 3H Medi Solution Co., Ltd., Tokyo 171-0022, Japan

**Keywords:** burden of caregivers, dementia with Lewy bodies, subanalysis, questionnaire survey

## Abstract

The burden of caregivers of people with dementia with Lewy bodies (DLB) is high; however, factors related to their caregiving burden are not fully clarified. We herein investigated factors associated with increasing caregiver burden for caregivers of people with DLB. To explore factors associated with caregiver burden, a linear regression analysis was conducted using the J-ZBI_8 total score as the dependent variable and a total of 36 factors as independent variables. This analysis included 252 pairs of people with DLB and their caregivers. Caregivers’ mean J-ZBI_8 was 8.4, indicating that caregiver burden was generally high. First, we identified 20 factors associated with caregiver burden in univariable analysis. Finally, multivariable analysis found three significant factors: irritability (β = 0.208, *p* < 0.001), use of “short stay” or “small-scale, multifunctional home care” (β = 0.208, *p* < 0.001), and nighttime behavior (β = 0.138, *p* = 0.020) were significantly associated with J-ZBI_8 total scores. Irritability and nighttime behavior were found to be contributing factors to caregiver burden. High caregiver burden among caregivers of people with DLB may result in the use of social services providing overnight stays, but to what extent such services reduce caregiver burden is unknown.

## 1. Introduction

The number of people with dementia is increasing as the aging population grows in Japan and other countries [1,2,3]. A cross-country analysis showed that dementia is currently the most prevalent medical condition that requires caregiving services [4]. The burden on the caregivers of people with dementia is very complex. Caregiving often requires taking on multiple duties to assist people with their activities of daily living (ADL) such as eating, dressing, mobility, and bathing. These caregiving burdens lead to a reduction in caregivers’ personal time and time to fulfill other roles, in addition to wider economic burdens such as cost of maintaining the social security system [5,6,7]. Due to the cognitive and functional decline associated with dementia, caregivers of people with dementia spend significantly more hours per week, and assist with more ADL and instrumental ADL (IADL), such as telephone use, shopping, housework, going out, and management of medication and money, on average than caregivers of people who do not have dementia [8].

Dementia with Lewy bodies (DLB) is the second most common degenerative dementia in old age after Alzheimer’s disease (AD) and is characterized by progressive cognitive impairment, along with a variety of clinical manifestations including psychiatric symptoms, rapid eye movement sleep behavior disorder (RBD), parkinsonism, and autonomic dysfunction [9,10,11,12]. Some of these clinical manifestations of DLB are expected to increase the burden of care for both caregivers and health and social care systems [13,14,15,16,17,18,19]. Additionally, when caregiver burden was examined based on the Zarit Burden Interview (ZBI), its short version (ZBI_8), or the Neuropsychiatric Inventory (NPI), the total caregiver distress scale was higher for caregivers of those with DLB than for caregivers of those with AD or vascular dementia [13,14,20].

Previous studies in people with DLB and other dementia disorders report that caregiver distress and burden are associated with their NPI score, psychosis (delusions, hallucinations, and agitation), mood disorder (anxiety, depression, and apathy), antidepressant drugs, daytime hypersomnia, eating behavior, Mini-Mental State Examination (MMSE) score, cognitive fluctuations, and impaired ADL and IADL [13,14,15,16,17,18]. However, these previous studies included caregivers of people with DLB and without DLB, and factors associated with the burden of caregivers for people with DLB have not been fully elucidated.

Although there is limited data specific to caregivers of people with DLB, previous studies did examine factors associated with caregiver burden among this population [19,20]. The study reported that psychosis, affection, and IADL were associated with caregiver burden; however, this was a retrospective single-center study and had potential bias in terms of the population included [19]. Furthermore, the above-mentioned studies focused mainly on psychiatric symptoms (Behavioral And Psychological Symptoms In Dementia [BPSD]), ADL, and cognitive function, and included only a small number of people with DLB [13,14,15,16,17,18,19,20]. These previous studies did not cover factors that are expected to influence the burden of care including clinical symptoms specific to DLB other than BPSD, ADL, and cognitive function; the use of social resources such as short-stay home care; and characteristics of the primary caregivers [13,14,15,16,17,18,19,20].

We conducted a questionnaire survey study in Japan to investigate the treatment needs of people with DLB and their caregivers, and their physicians’ awareness of those treatment needs, the findings of which have already been published [21]. Here, we conducted an exploratory subanalysis to examine the factors associated with caregiver burden including the severity and presence of clinical symptoms, the use of social resources by people with DLB, and the relationship between the caregivers and the attending physicians using the data collected from the main study.

## 2. Materials and Methods

### 2.1. Study Design and Participants

This was an exploratory subanalysis of a multicenter, cross-sectional, observational, questionnaire-based survey study conducted in Japan at 35 study sites that employed physicians with expertise in DLB [21]. The main study was conducted from September 2020 to July 2021.

The study participants were people with DLB, their caregivers, and their attending physicians. People with DLB were included if they had a diagnosis of probable DLB [11], were aged ≥50 years, had an attending physician that had been in practice for >3 months, were outpatients, and had a caregiver. Probable DLB was diagnosed if people who had a progressive cognitive decline of sufficient magnitude to interfere with normal social or occupational functions or usual daily activities met either of the following criteria: (1) two or more core clinical features of DLB (fluctuating cognition, recurrent visual hallucinations, REM sleep behavior disorder, or parkinsonism [bradykinesia, rest tremor, or rigidity]) were present with or without the presence of indicative biomarkers; or (2) only one core clinical feature was present with one or more indicative biomarkers [11]. Patients with Parkinson’s disease with dementia (if parkinsonism had been present for more than 1 year prior to the onset of dementia); patients whose attending physician had not seen them for more than 3 months prior to obtaining consent; and institutionalized patients were excluded. Caregivers were included if they were ≥20 years old and were the primary caregiver of the people with DLB. Attending physicians had to be experts in DLB treatment in Japan; a detailed definition was provided in the main study paper [21].

The contents of the questionnaire given to the people with DLB (PQ), caregivers (CQ), and attending physicians (PhQ); the survey methodology; and definitions for each symptom of DLB were provided in the main study paper [21].

### 2.2. Assessments

People with DLB and caregivers underwent a variety of assessments. Caregiver burden was assessed using the short version of the Japanese version of the ZBI_8 (J-ZBI_8), which has been validated and is considered a reliable instrument to measure caregiver burden [14,22,23]. BPSD and cognitive fluctuations of people with DLB were assessed by caregivers using the Japanese version of the NPI-12 [24] and the Cognitive Fluctuation Inventory (CFI) [25]. The degree of cognitive impairment was assessed using the Japanese version of the MMSE (MMSE-J) [26]. ADL and the severity of parkinsonism were assessed using the Japanese version of the Movement Disorder Society–Unified Parkinson’s Disease Rating Scale (MDS–UPDRS) Parts II and III, respectively [27].

### 2.3. Statistical Analysis

Descriptive statistics were used to evaluate participants’ background characteristics. Summary statistics were calculated as mean ± standard deviation (SD) for continuous variables and frequency and percentage for categorical variables.

To explore factors associated with the caregiver burden, linear regression analysis with the forced-entry method was conducted using the J-ZBI_8 total score as the dependent variable and a total of 36 factors as independent variables. Multivariable analysis was conducted with factors that were significant in the univariable analysis. The 36 factors included 5 items related to characteristics of people with DLB, 18 to the clinical status of people with DLB, 8 to caregiver characteristics, and 5 to other factors.

The five items related to characteristics of people with DLB were (1) age, (2) sex, (3) number of months since diagnosis of DLB, (4) number of years of education, and (5) knowledge of DLB [21].

The 18 items related to the clinical status of people with DLB were as follows: the MMSE-J total score, CFI score, MDS–UPDRS Part II total score, MDS–UPDRS Part III total score, NPI-12 subitem scores, presence of autonomic dysfunction, and presence of sensory disorders.

The eight items related to the caregiver characteristics were as follows: (1) caregiver’s age, (2) caregiver’s sex, (3) caregiver’s knowledge of DLB, (4) whether the caregiver lived with the people with DLB, (5) hours spent with the people with DLB each day, (6) caregiver’s employment status, (7) relationship with the people with DLB from the perspective of the people with DLB, and (8) presence of assistant caregivers.

The five other factors were as follows: (1) frequency of hospital or clinic visits, (2) use of “long-term care (daytime service)” or “outpatient rehabilitation (daytime care)”, (3) use of “short stay” or “small-scale, multifunctional home care”, (4) from the caregiver’s perspective, how well the attending physician listens to what the caregiver says, and (5) from the caregiver’s perspective, whether there is someone at the hospital or clinic other than the attending physician with whom the caregiver can talk.

Of note, “small-scale, multifunctional home care” refers to a community-based, in-home nursing care service that offers care services for older people who require nursing care and who live in their own homes. This type of care service was established in Japan in response to the rapidly aging population in urban areas and provides a combination of in-home services, outpatient day long-term service, and short-term stays. Since small-scale multifunctional home care provides overnight services, it was analyzed in the same category as a short stay in this study.

NPI-12 subitems were classified as with symptoms (≥1 point) or without symptoms (0 point), and factors for other continuous variables were classified as above or below the median value. The multivariable model used a forced-entry method.

Missing data were excluded from the analysis on the basis of missing items. In the multivariable analysis, if a variable with missing data was included in the analysis model, it was excluded on a case-by-case basis.

The statistical significance level in this study was set at 0.05 (two-sided); all analyses were conducted using Statistical Analysis Software version 9.4 (SAS Institute Inc., Cary, NC, USA).

## 3. Results

### 3.1. Participants

**Figure 1:**
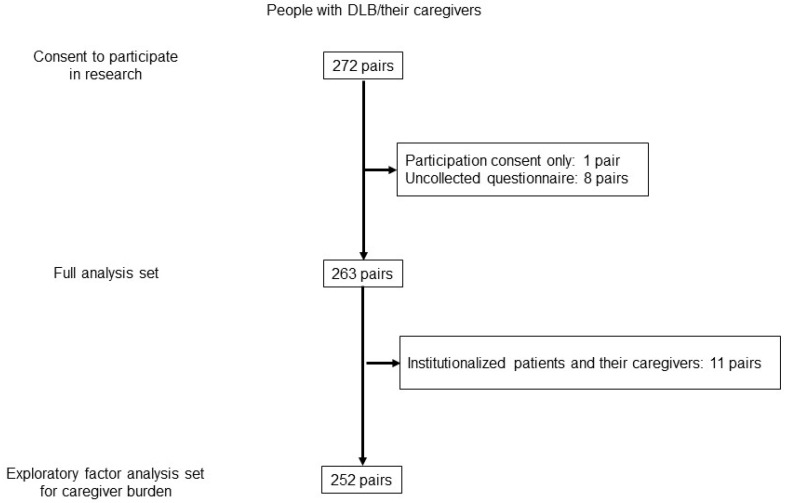
Flowchart of the study participants.

Among the 263 pairs of people with DLB and their caregivers in the full analysis set of the main study, 11 pairs were excluded due to patient institutionalization. Therefore, 252 pairs were included in the analysis population for the present subanalysis (Figure 1).

Background characteristics are summarized in Table 1. People with DLB had a mean (SD) age of 79.1 (6.7) years, 49.2% were male, and the mean (SD) duration after diagnosis of DLB was 30.2 (30.1) months. The proportions of people who used care services were 49.6% for “long-term care” or “outpatient rehabilitation”, and 21.0% for “short stay” or “small-scale, multifunctional home care”. The mean (SD) MMSE-J total score was 21.0 (5.8); NPI-12 total score, 15.8 (16.0); and MDS–UPDRS Part III total score, 23.7 (20.8). The frequency of parkinsonism was 74.2% based on the assessment toolkits for the diagnosis of DLB [28]. Caregivers had a mean (SD) age of 65.1 (12.9) years, most were female (72.2%), and the most common relationship with the people with DLB was spouse (55.2%). Most caregivers (83.3%) lived with the people with DLB, with the mean (SD) time per day spent together being 15.0 (8.6) h. The mean (SD) J-ZBI_8 total score was 8.4 (6.5).

### 3.2. Factors Associated with Caregiver Burden

The results of the univariable and multivariable analysis of factors associated with caregiver burden, as determined by the J-ZBI_8 total score, are shown in Table 2 and Table 3. Univariable analysis using the J-ZBI_8 total score as a dependent variable showed that the following 20 factors were significantly associated with caregiver burden: age of the people with DLB, number of months since diagnosis of DLB, frequency of hospital or clinic visits, use of “long-term care” or “outpatient rehabilitation”, use of “short stay” or “small-scale, multifunctional home care”, MMSE-J total score, MDS–UPDRS Part II total score, and all subdomains of the NPI-12 (delusions, hallucinations, agitation, depression, anxiety, apathy, disinhibition, irritability, aberrant motor behavior, nighttime behavior, and appetite) except for euphoria, CFI score, and assistant caregiver status (Table 2).

Multivariable analysis of these 20 items revealed that irritability (β = 0.208, *p* < 0.001), use of “short stay” or “small-scale, multifunctional home care” (β = 0.208, *p* < 0.001), and nighttime behavior (β = 0.138, *p* = 0.020) were significantly associated with J-ZBI_8 total scores (Table 3).

## 4. Discussion

This exploratory subanalysis examined factors associated with caregiver burden, as determined by the J-ZBI_8 total score in people with DLB, using a dataset from a multicenter, cross-sectional, observational, questionnaire-based survey [21]. This subanalysis examined the association of various factors (a total of 36 factors) with caregiver burden, and the univariable analysis revealed that 20 factors were significantly associated with caregiver burden, which is generally consistent with the findings of previous studies on people with DLB and other dementia disorders [13,14,15,16,17,18,19,20]. Notably, only three factors (irritability, nighttime behavior, and use of “short stay” or “small-scale, multifunctional home care”) were identified as significant factors in the multivariable analysis. To the best of our knowledge, this is the first study to simultaneously explore a large range of factors and assess their effects on caregiver burden specific to DLB, and to report that nighttime behavior increases caregiver burden.

In a previous study examining factors associated with caregiver burden among people with dementia, irritability was also found to be a contributing factor to caregiver burden [14]. In another previous study that included only caregivers of people with DLB and used the NPI total caregiver distress scale, behavioral and emotional problems such as irritability, among other factors, were found to be significantly associated with caregiver burden [13]. Thus, our findings are generally consistent with the results of previous studies of people with dementia including DLB [13,14]. The prevalence of irritability was not high (34.8%) compared with other BPSD in the present study, but irritability is a symptom that requires early diagnosis and treatment to reduce caregiver burden.

This study included a large number of people with mild DLB, which highlights that nighttime behavior is a significant caregiver burden in DLB even from the mild stage of the disease. The clinical features of DLB include various sleep-related disorders such as abnormal daytime sleepiness [29], nocturnal sleep disturbance [30], RBD, and restless legs syndrome. In particular, RBD and hypersomnia are frequent symptoms in people with DLB [31] and are used as diagnostic criteria for DLB [17]. This subanalysis did not examine which types of sleep-related disorders affected caregiver burden. However, the main study reported that nighttime dysuria, RBD, insomnia, and hypersomnia were frequent symptoms that caused caregivers the most distress [21], suggesting that nighttime dysuria and insomnia, as well as RBD and hypersomnia, may also increase caregiver burden. People with DLB with urinary disorders may require significant assistance with the elimination of bodily waste. In fact, a previous survey of 962 caregivers of people with DLB showed that up to 65% of people with DLB required assistance with body waste elimination [18]. Furthermore, the previous study reported that caregiver burden based on the NPI-D score for sleep disturbances was higher for the people with DLB and their caregivers than for people with AD and their caregivers [32]. Therefore, nighttime behavior or sleep-related disorders including nighttime dysuria may place a high burden on caregivers of people with DLB, leading to the use of “short stay” or “small-scale, multifunctional home care” facilities. Although hypnotic agents are used to improve the quality of sleep, they may have adverse effects such as daytime sleepiness, confusion, amnesia, and increased frequency of falling [24,33,34]. Importantly, the results of this study show that sleep-related disorders are still a burden for caregivers, despite the availability of pharmacologic and non-pharmacologic treatments for the disorder. Considering that sleep disturbances in people with DLB place a high burden on caregivers—as well as on health and social care systems—there is a need for management to prevent daytime sleepiness and enable regular daily nighttime sleep according to the cause of the sleep-related disorders, including both pharmaco- and non-pharmaco-therapies [35].

Hallucination and delusion have been reported as a cause of high caregiver burden in previous studies [16,19]. In the present study, all NPI-12 subitems except euphoria were significantly associated with caregiver burden in univariable analysis, but after adjustment by multivariable analysis, all subitems other than irritability and nighttime behavior did not reach statistical significance. One possible reason for this is that this study included people with DLB with mild or moderate BPSD such as hallucinations or delusions. In such a population, irritability and nighttime behavior may be identified as statistically significant factors associated with increased caregiver burden. Moreover, it is well known that visual hallucination is more frequent in the evening and during the night [36]. Therefore, more attention should be paid to sleep disturbances and irritability as well as hallucinations and delusions from the viewpoint of caregiver burden in DLB, especially in those with DLB with mild or moderate severity of BPSD such as hallucination and delusion.

There have been limited reports examining the association between caregiver burden and social welfare care services in people with DLB. This subanalysis found that the use of “long-term care” or “outpatient rehabilitation”, use of “short stay” or “small-scale, multifunctional home care”, and the presence of an assistant caregiver were associated with caregiver burden in the univariable analysis. Furthermore, the multivariable analysis identified the use of “short stay” or “small-scale, multifunctional home care” as a factor related to caregiver burden. The use of these services and of assistant caregivers were assumed to reduce caregiver burden, but caregiver burden was higher in people with DLB who used these services compared with those who did not in the present study. This study did not evaluate changes in caregiver burden before and after service use; therefore, our results do not necessarily indicate that the use of “short stay” or “small-scale, multifunctional home care” increases the caregivers’ burden. Furthermore, it was previously reported that, among individuals with dementia, those with neuropsychiatric symptoms required significantly more active help or supervision by an informal caregiver than those with no neuropsychiatric symptoms [37]. Not having to care for people with DLB, even if for a short period of time, may allow caregivers to spend time on leisure activities (hobbies and recreational activities) and temporarily reduce the burden and stress of caregiving [38]. Therefore, we speculate that people with a high care burden, such as the people with DLB in the present analysis, tend to use “short stay” or “small-scale, multifunctional home care” services. Longitudinal studies are needed to determine whether these services reduce the caregiver burden.

Parkinsonism is one of the core clinical features of DLB [11], and 74.2% of people with DLB had parkinsonism in this study. In general, parkinsonism increases caregiver burden, but the univariable analysis showed no significant association between the severity of parkinsonism and caregiver burden. This is consistent with the results of the main study: parkinsonism was the most frequent symptom that caused the people with DLB the most distress, but this was not the case for their caregivers. This study was conducted with people with DLB living at home, suggesting that many of them had relatively mild parkinsonism. For patients with Parkinson’s disease, the cut-off points for Parkinson’s disease severity based on the MDS–UPDRS Part III have been reported as 32/33 for mild/moderate and 58/59 for moderate/severe [39]. Furthermore, in the recent clinical trial of patients with DLB who required treatment of parkinsonism, the mean total score of the MDS–UPDRS Part III was 40.2 [40]. In comparison, the MDS–UPDRS Part III scores in this study were notably low (median total score, 18), supporting the notion that most had mild parkinsonism. In such a population, parkinsonism may place less of a burden on caregivers.

This study had some limitations. First, the level of caregiver burden was assessed using the simplified version of the J-ZBI_8. If we had conducted an analysis of factors that increase the level of caregiver burden using the standard version of the J-ZBI, other than irritability and sleep disturbance, we may have been able to extract BPSD and cognitive impairment, which have been reported in previous studies as factors contributing to caregiver burden [15,41]. Second, the people with DLB who participated in this study were outpatients whose attending physicians determined that many of them had mild DLB symptoms and were able to complete the questionnaire. As such, these results do not apply to inpatients or people with severe DLB. Third, this study included people with probable DLB according to the fourth consensus report of the DLB Consortium [11], but did not collect information on whether instrumental exams (e.g., DAT-SPECT) were performed in all patients. Fourth, this study was based on a questionnaire survey, which has inherent limitations, as previously described in the main study [21]. Fifth, this study had limitations regarding type I error; we did not adjust for multiplicity by repeating the test for the 36 factors due to the sample size in the univariable analysis. Finally, this study was conducted during the COVID-19 pandemic, a period of much uncertainty, unprecedented challenges, and restrictions worldwide, which may have increased the level of stress for caregivers [42].

Despite these limitations, this study has several strengths including that it was a multicenter study with a relatively large sample size, thereby limiting bias in participant selection. Additionally, reliable diagnoses were obtained by physicians with expertise in DLB. Furthermore, this study also examined factors such as the use of social resources and characteristics of primary caregivers. Accordingly, we consider the three factors identified in this study—irritability, nighttime behavior, and use of “short stay” or “small-scale, multifunctional home care—to be core factors associated with caregiver burden in people with DLB.

## 5. Conclusions

This study aimed to explore the factors associated with increased caregiver burden specific to caregivers of people with DLB. Irritability and nighttime behavior in people with DLB were found to be contributing factors to caregiver burden. It is also suggested that people with DLB with high caregiving burden use social services that provide overnight stays. Further studies such as interventional and longitudinal studies are required to confirm whether improvements in irritability and nighttime behavior and the use of these services can reduce the burden on caregivers.

## Figures and Tables

**Table 1:** Background characteristics of people with dementia with Lewy bodies and their caregivers.

	*N* = 252
Age of people with DLB (years)	79.1 ± 6.7
Sex of people with DLB (male)	124 (49.2)
Duration after diagnosis of DLB (months)	30.2 ± 30.1 (*n* = 249)
Education (years)	11.8 ± 2.9 (*n* = 233)
Institute University hospital Non-university hospital Clinic	117 (46.4) 49 (19.4) 86 (34.1)
Number of people living with the people with DLB at their own home (*n* = 245) Alone Two or more	25 (10.2) 220 (89.8)
Use of “long-term care” or “outpatient rehabilitation” (yes)	125 (49.6)
Use of “short stay” or “small-scale, multifunctional home care” (yes)	53 (21.0)
MMSE-J total score	21.0 ± 5.8
Neuropsychiatric Inventory-12 total score	15.8 ± 16.0 (*n* = 250)
Delusions (Yes) Hallucinations (Yes) Agitation (Yes) Depression (Yes) Anxiety (Yes) Euphoria (Yes) Apathy (Yes) Disinhibition (Yes) Irritability (Yes) Aberrant motor behavior (Yes) Nighttime behavior (Yes) Appetite (Yes)	76 (30.4) 120 (48.0) 76 (30.4) 97 (38.8) 100 (40.0) 27 (10.8) 121 (48.4) 30 (12.0) 87 (34.8) 51 (20.4) 90 (36.0) 69 (27.6)
MDS–UPDRS Part III total score	23.7 ± 20.8 (*n* = 250)
MDS–UPDRS Part II total score	11.2 ± 10.9 (*n* = 251)
Cognitive Fluctuation Inventory score	2.2 ± 3.0
Autonomic dysfunction (yes)	106 (42.1)
Sensory disorder (yes)	26 (10.3)
Caregiver’s age (years)	65.1 ± 12.9
Caregiver’s sex (male)	70 (27.8)
Caregiver’s relationship with the people with DLB Spouse Non-spouse	139 (55.2) 113 (44.8)
Caregiver lives with the people with DLB (yes)	210 (83.3)
Caregiver’s time spent with the people with DLB (hours/day)	15.0 ± 8.6 (*n* = 247)
Caregiver is concurrently employed (yes)	102 (40.8)
Assistant caregiver (yes)	87 (34.5)
J-ZBI_8 total score	8.4 ± 6.5 (*n* = 252)

Data are mean ± standard deviation or *n* (%). Abbreviations: J-ZBI_8, the Japanese version of the Zarit Caregiver Burden Interview 8; MDS–UPDRS, Movement Disorder Society–Unified Parkinson’s Disease Rating Scale; MMSE-J, Mini-Mental State Examination.

**Table 2:** Univariable analysis ^a^ of the J-ZBI_8 total score.

Factors		*n*	Mean ± SD	*p*-Value
Age of people with DLB (years)	<80.0 ≥80.0	117 135	7.48 ± 5.91 9.11 ± 6.83	0.046
Sex of people with DLB	Female Male	128 124	7.93 ± 6.15 8.78 ± 6.77	0.297
Duration since diagnosis of DLB (months)	<24.0 ≥24.0	121 128	7.35 ± 5.77 9.21 ± 6.92	0.022
Education level of people with DLB (years)	<12.0 ≥12.0	73 160	7.96 ± 6.87 8.33 ± 6.15	0.688
DLB knowledge level of people with DLB	A lot Neither yes nor no, does not know very much	18 231	5.61 ± 5.54 8.62 ± 6.49	0.057
Frequency of hospital or clinic visits	1. Once every 2 to 3 weeks, once every month 2. Once every 2 months, once every 3 months 3. Once every 4 months or more	100 143 3	10.08 ± 6.59 7.23 ± 6.17 7.33 ± 2.08	0.001 (1 vs. 2) 0.459 (1 vs. 3) 0.978 (2 vs. 3)
Use of “long-term care” or “outpatient rehabilitation”	None Yes	127 125	6.72 ± 5.89 10.00 ± 6.62	<0.001
Use of “short stay” or “small-scale, multifunctional home care”	None Yes	199 53	7.27 ± 5.77 12.42 ± 7.30	<0.001
MMSE-J total score	≥22 <22	130 122	6.70 ± 5.99 10.11 ± 6.51	<0.001
MDS–UPDRS Part II total score	<9 ≥9	124 127	6.99 ± 5.75 9.74 ± 6.82	0.001
MDS–UPDRS Part III total score	<18 ≥18	122 128	7.66 ± 6.05 9.10 ± 6.78	0.080
Neuropsychiatric Inventory-12 subitems				
Delusions	None Yes	174 76	7.10 ± 6.03 11.25 ± 6.60	<0.001
Hallucinations	None Yes	130 120	6.42 ± 5.52 10.47 ± 6.81	<0.001
Agitation	None Yes	174 76	6.75 ± 5.89 12.05 ± 6.31	<0.001
Depression	None Yes	153 97	7.43 ± 6.21 9.83 ± 6.67	0.004
Anxiety	None Yes	150 100	7.39 ± 6.20 9.82 ± 6.65	0.003
Euphoria	None Yes	223 27	8.10 ± 6.53 10.48 ± 5.80	0.072
Apathy	None Yes	129 121	6.23 ± 5.70 10.63 ± 6.53	<0.001
Disinhibition	None Yes	220 30	7.99 ± 6.45 11.10 ± 6.16	0.013
Irritability	None Yes	163 87	6.63 ± 5.74 11.61 ± 6.58	<0.001
Aberrant motor behavior	None Yes	199 51	7.41 ± 6.08 12.06 ± 6.75	<0.001
Nighttime behavior	None Yes	160 90	6.82 ± 5.70 11.10 ± 6.91	<0.001
Appetite	None Yes	181 69	7.65 ± 6.33 10.23 ± 6.56	0.005
Cognitive Fluctuation Inventory score	0 ≥1	109 143	6.14 ± 5.80 10.04 ± 6.45	<0.001
Autonomic dysfunction	None Yes	146 106	7.84 ± 6.33 9.06 ± 6.60	0.138
Sensory disorder	None Yes	226 26	8.25 ± 6.50 9.23 ± 6.21	0.464
Caregiver’s age (years)	<65 ≥65	121 131	8.88 ± 6.39 7.86 ± 6.51	0.213
Caregiver’s sex	Female Male	182 70	8.59 ± 6.53 7.73 ± 6.27	0.345
Caregiver’s knowledge of DLB	A lot Neither yes nor no, does not know very much	63 184	9.35 ± 6.44 8.08 ± 6.40	0.117
Physician listens to what the caregiver says from the caregiver’s perspective	1. Very well, well 2. Normal 3. Not much, not at all, does not know	217 25 10	8.43 ± 6.31 6.80 ± 7.09 10.60 ± 7.88	0.234 (1 vs. 2) 0.298 (1 vs. 3) 0.117 (2 vs. 3)
Anyone other than the physician with whom the caregiver can talk in hospital or clinic	1. Yes 2. None 3. Do not know	98 126 26	8.82 ± 6.88 7.86 ± 5.86 8.69 ± 7.24	0.268 (1 vs. 2) 0.929 (1 vs. 3) 0.547 (2 vs. 3)
Caregiver lives with the people with DLB	Yes None	210 42	8.47 ± 6.33 7.74 ± 7.14	0.507
Caregiver’s time spent with the people with DLB (hours/day)	<16.0 ≥16.0	112 135	8.52 ± 6.59 8.42 ± 6.39	0.906
Caregiver is concurrently employed	Yes None	102 148	8.62 ± 6.92 8.16 ± 6.16	0.584
Caregiver’s relationship with the people with DLB	Spouse Non-spouse	139 113	8.17 ± 6.46 8.57 ± 6.49	0.630
Assistant caregiver	Yes None, unknown	87 165	10.07 ± 7.03 7.44 ± 5.96	0.002

^a^ Univariable analysis with 36 independent variables and the J-ZBI_8 total score as the dependent variable. Abbreviations: DLB, dementia with Lewy bodies; J-ZBI_8, the Japanese version of the Zarit Caregiver Burden Interview 8; MMSE-J, Mini-Mental State Examination Japanese version; MDS–UPDRS, Movement Disorder Society–Unified Parkinson’s Disease Rating Scale; SD, standard deviation.

**Table 3:** Multivariable analysis of the J-ZBI_8 total score.

Factors	B	SE	β	*t*-Statistic	*p*-Value	VIF
Age of people with DLB (years)						
<80	ref					
≥80	0.559	0.727	0.043	0.769	0.443	1.197
Duration after diagnosis of DLB (months)						
<24	ref					
≥24	0.697	0.748	0.054	0.931	0.353	1.273
Frequency of hospital or clinic visits						
Once every 2 to 3 weeks, once every month	ref					
Once every 2 months, once every 3 months	−1.439	0.743	−0.110	−1.938	0.054	1.209
Once every 4 months or more	−2.092	3.095	−0.036	−0.676	0.500	1.076
Use of “long-term care” or “outpatient rehabilitation”						
None	ref					
Yes	0.969	0.770	0.075	1.258	0.210	1.349
Use of “short stay” or “small-scale, multifunctional home care”						
None	ref					
Yes	3.278	0.985	0.208	3.327	**<0.001**	1.479
MMSE-J total score						
≥22	ref					
<22	0.302	0.787	0.023	0.383	0.702	1.409
MDS–UPDRS Part II total score						
<9	ref					
≥9	0.493	0.764	0.038	0.646	0.519	1.327
Neuropsychiatric Inventory-12 subitems						
Delusions						
None	ref					
Yes	1.569	0.919	0.113	1.708	0.089	1.637
Hallucinations						
None	ref					
Yes	1.190	0.799	0.092	1.489	0.138	1.452
Agitation						
None	ref					
Yes	0.338	0.893	0.024	0.378	0.705	1.524
Depression						
None	ref					
Yes	−0.401	0.766	−0.030	−0.523	0.602	1.273
Anxiety						
None	ref					
Yes	0.650	0.787	0.049	0.826	0.410	1.348
Apathy						
None	Ref					
Yes	0.748	0.778	0.058	0.961	0.337	1.375
Disinhibition						
None	Ref					
Yes	−0.639	1.118	−0.032	−0.572	0.568	1.208
Irritability						
None	Ref					
Yes	2.795	0.819	0.208	3.413	**<0.001**	1.396
Aberrant motor behavior						
None	Ref					
Yes	1.128	0.957	0.071	1.178	0.240	1.376
Nighttime behavior						
None	Ref					
Yes	1.852	0.790	0.138	2.345	**0.020**	1.305
Appetite						
None	Ref					
Yes	0.532	0.805	0.037	0.660	0.510	1.177
Cognitive Fluctuation Inventory score						
0	ref					
≥1	1.378	0.785	0.106	1.756	0.080	1.373
Assistant caregiver						
Yes	ref					
None	−0.659	0.776	−0.049	−0.849	0.397	1.233

*p*-values in bold font indicate statistical significance. Abbreviations: β, standard regression coefficient; B, regression coefficient; J-ZBI_8, the Japanese version of the Zarit Caregiver Burden Interview 8; MDS–UPDRS, Movement Disorder Society–Unified Parkinson’s Disease Rating Scale; MMSE-J, Mini-Mental State Examination-Japanese; ref; reference, SE, standard error; VIF, variance inflation factor.

## Data Availability

The datasets presented in this article are not readily available due to participant privacy. Requests to access the datasets should be directed to the corresponding author.
